# The effect of heterobifunctional crosslinkers on HEMA hydrogel modulus and toughness

**DOI:** 10.1371/journal.pone.0215895

**Published:** 2019-05-09

**Authors:** Elizabeth M. Boazak, Vaughn K. Greene, Debra T. Auguste

**Affiliations:** 1 Department of Biomedical Engineering, The City College of New York, New York, New York, United States of America; 2 Department of Chemical Engineering, Northeastern University, Boston, Massachusetts, United States of America; University of Maryland Baltimore County, UNITED STATES

## Abstract

The use of hydrogels in load bearing applications is often limited by insufficient toughness. 2-Hydroxyethyl methacrylate (HEMA) based hydrogels are appealing for translational work, as they are affordable and the use of HEMA is FDA approved. Furthermore, HEMA is photopolymerizable, providing spatiotemporal control over mechanical properties. We evaluated the ability of vinyl methacrylate (VM), allyl methacrylate (AM), and 3-(Acryloyloxy)-2-hydroxypropyl methacrylate (AHPM) to tune hydrogel toughness and Young’s modulus. The crosslinkers were selected due to their heterobifunctionality (vinyl and methacrylate) and similar size and structure to EGDMA, which was shown previously to increase toughness as compared to longer crosslinkers. Vinyl methacrylate incorporation into HEMA hydrogels gave rise to hydrogels with Young’s moduli spanning ranges for ligament to cartilage, with a peak toughness of 519 ± 70 kJ/m^3^ under physiological conditions. We report toughness (work of extension) as a function of modulus and equilibrium water content for all formulations. The hydrogels exhibited 80%-100% cell viability, which suggests they could be used in tissue engineering applications.

## Introduction

Hydrogels, crosslinked polymer networks that absorb water, are widely used for biomedical devices, drug delivery, implants, and tissue engineering [1]. Hydrogel mechanical properties are modified principally by varying macromer molecular weight, and macromer, initiatior, and crosslinker concentrations. The cross-linking density of the gel network is proportional to the gel's elastic modulus and inversely proportional to its swelling [2]. High degrees of swelling are often desired for improved transport within the hydrogel. However, swelling reduces not only material stiffness, but also toughness. Inherently low stiffness and toughness has limited the use of hydrogels in load bearing applications [3]. Crosslinking density is also related to mesh (or pore) size, which impacts cell differentiation, viability, and migration [4–6]. Previous reports have optimized hydrogel mechanics for specific gene regulation [7]. As such, the development of tough hydrogels with tunable moduli and swelling properties remains a challenge.

Toughness, also known as work of extension, is described by the area under the stress-strain curve until failure, giving units of energy/volume. This is a measure of energy that includes fracture energy, elastically stored energy, and energy dissipated through plastic deformation [8]. Toughness is correlated with wear resistance in implants, meaning that it also reflects material durability [9, 10]. Attempts to improve hydrogel toughness have included homogeneous gels, slip-link gels, double-network gels, nanocomposite gels and gels formed using poly-functional crosslinkers [8]. Double-network approaches have yielded gels with high swelling ratios and toughness, ranging from 10^3^–10^5^ J m^-3^ [8, 11]. This is in contrast to the 10–10^3^ J m^-3^ range reported for conventional hydrogels [8, 12]. Recently, high stiffness and high toughness has been achieved with poly-anion complex hydrogels, though biocompatibility remains to be established [13].

Double network hydrogels can approach the toughness of native tissues. The trachea is estimated to have a composite longitudinal toughness of 85–240 kJ m^-3^ [14, 15]; said longitudinal deformation and its associated toughness primarily reflect the properties of ligament and smooth muscle. Meanwhile, tendon toughness ranges from 1000 to 5000 kJ m^-3^[16, 17]. Comparison of hydrogel toughness to cartilage toughness has been complicated by differences between the mechanical tests commonly used to evaluate the two materials; while hydrogels here and elsewhere are frequently assessed under tension, and toughness assessed as the area under the stress vs. strain field, cartilage and bone are more frequently evaluated under compressive loads, with reported toughness values referring to fracture toughness [18].

Despite leading to large gains in hydrogel toughness, the aforementioned strategies are still unable to replicate the toughness of many biological tissues, and have limitations with regards to modulus and spatiotemporal tunability. The highest moduli for double network gels is below 1 MPa, making them unable to replicate the mechanical properties of stiffer tissues like cartilage and bone. The tensile modulus reported for human cartilage ranges from 1–20 MPa [19, 20]. Under quasi-static/constant-rate loading, a tensile modulus of 34.5 MPa has been reported [21]. Bone is approximately three orders of magnitude higher, ranging from 1–20 GPa [22, 23]. Materials with low modulus may facilitate cell differentiation, but are not suitable for applications where the ability to bear loads is required upon implantation, such as in tracheal replacement [15]. Furthermore, homogeneous, slip-link, and double-network hydrogels all increase toughness via mechanisms that limit tunability and/or prevent spatial patterning, as can be achieved with UV photopolymerization of conventional hydrogels. Tunability and spatiotemporal control are particularly important for applications requiring higher-level structure. Gradients in mechanical properties will likely be required to address injuries affecting the connection between ligaments or tendons and bone, or replicating the interface between cartilage and bone [24].

Prior use in FDA approved products and affordability make HEMA a particularly attractive hydrogel for translational work. HEMA hydrogels with a variety of formulations and techniques are reported in the literature with Young’s moduli ranging from <100 kPa [25] to 1.5 GPa [26]. In addition, we produced spatially patterned HEMA hydrogels with high and low modulus regions ranging from 67 kPa to 13 MPa [27]. The combination of material properties and hydrogel architecture yielded mechanical properties that mimicked that of neonatal trachea [27]. However, HEMA hydrogels decrease in modulus and fail in a brittle manner under fully hydrated conditions. A tunable material with similar moduli but greater toughness is needed.

Crosslinker chemistry was demonstrated to modulate bulk material properties. Recent work by Moghadam et al. indicated that HEMA hydrogels crosslinked with EGDMA are tougher than those crosslinked with TEGDMA [3]; the similar length of EGDMA to HEMA side chains may increase reversible hydrophobic interactions. However, elastic moduli were not reported for these gels. Separately, copolymers of methyl methacrylate and methyl acrylate were described to have high toughness [10]. Generally acrylates are more flexible and extensible polymers, while methacrylates are stiffer and more rigid. Drawing inspiration from the mechanical advantages of copolymer networks and EGDMA crosslinker length, we hypothesized that using crosslinkers of similar length with reactive groups of varied structure may give rise to desirable mechanical properties.

**Fig 1:**
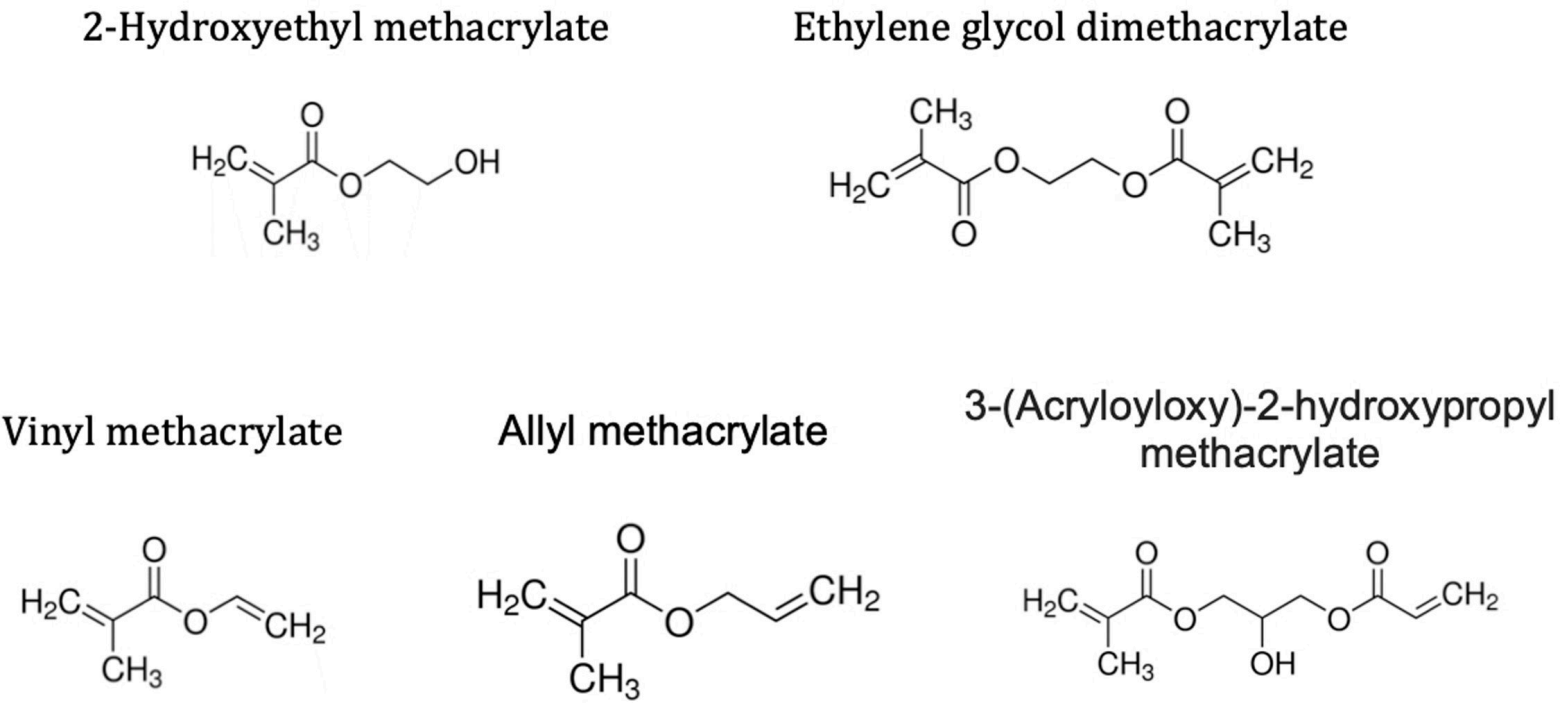
Chemical structures of HEMA and crosslinkers EGDMA, VM, AM, and AHPM.

In this work, we synthesized HEMA hydrogels with different crosslinkers and compared their mechanical properties to those co-crosslinked with EGDMA and EGDMA alone. We selected vinyl methacrylate (VM), allyl methacrylate (AM), and 3-(Acryloyloxy)-2-hydroxypropyl methacrylate (AHPM) due to their similar size and structure to EGDMA (**Fig 1**) and heterobifunctionality (vinyl and methacrylate). The goal of this work was to identify a formulation for tough, photopolymerizable HEMA-based hydrogels with a modulus tunable over a 50 kPa to 10 MPa range to encompass the moduli of cartilage and softer musculoskeletal tissues like ligament and smooth muscle [28].

## Materials and methods

### Materials

2-Hydroxyethyl methacrylate (HEMA) was purchased from TCI Chemicals (Montgomeryville, PA). Ethylene glycol dimethacrylate (EGDMA), vinyl methacrylate (VM), 3-(Acryloyloxy)-2-hydroxypropyl methacrylate (AHPM), allyl methacrylate (AM), and 2,20-Azobis (2-methylpropionitrile) (AIBN) were purchased from Sigma Aldrich (St. Louis, MO). Materials were used as received. Human neonatal dermal fibroblasts were purchased from Cascade Biologics (Portland, OR).

### Hydrogel synthesis

Prepolymer solutions were prepared by sequentially adding HEMA, crosslinker, ultra pure water (UPW), and AIBN to a 50 ml conical tube and inverting it several times to combine. The molar ratio of HEMA:crossliner:UPW was kept constant at 12.5:4.2:26.3, while the crosslinker composition was varied according to the ratios in **Table 1**. The prepolymer solution was degassed for 20 minutes and then injected between two glass slides separated by and 3.4 mm rubber spacer using a needle attached to a syringe. During injection, a secondary needle was inserted through the rubber spacer to allow air to be displaced from the mold interior. The rubber spacer was held in place with binder clips along the slide perimeter. This mold was then placed 0.5 inches above the bottom of a 1.5 inch deep tray containing a sufficient volume of room temperature tap water to cover the top surface of the mold. The water bath promoted even polymerization of the hydrogel sheets, and prevented overheating and cracking. The hydrogels were then polymerized by UV heating, which enabled consistent cross linking through the depth of the hydrogel, under two 8 W, 368 nm UV bulbs placed 1 inch above the gel surface. (Eiko, F8T5). (The use of Irgacure 2959, a photoinitiator, was also investigated, however, the induced polymerization resulted in excessive heat generation and cracking.) Polymerization time was varied to investigate the full range of moduli achievable for each polymer formulation. After synthesis, hydrogel sheets were placed in 10 cm dishes and hydrated in phosphate buffered saline (PBS) for 1 week on a shaker table.

**Table 1:** All hydrogels were synthesized with a 12.5/4.2/26.3 molar ratio of HEMA/crosslinker/UPW, while the chemical crosslinker or ratio of crosslinkers was varied.

Name	Molar Ratio EGDMA/Crosslinker
VM only	0/4.2
EGDMA/VM	1/3.2
EGDMA/VM 1.4/2.8	1.4/2.8
EGDMA/VM 3.2/1	3.2/1
AM only	0/4.2
EGDMA/AM	1/3.2
AHPM only	0/4.2
EGDMA/AHPM	1/3.2

### Mechanical testing

Rectangular samples approximately 30 mm in length and 5 mm in width were cut from 3mm thick hydrogel sheets and evaluated under tensile loading at 1 mm/min using an Instron 5543 mechanical testing system and either a 10N or 500N load cell. Initial sample dimensions were measured with calipers. Sandpaper was adhered to the pneumatic grips in order to reduce sample slipping. All samples were tested under physiological conditions, i.e. 37°C in a PBS, by submerging the clamped samples in the Instron BioPuls temperature controlled bath. Sample dimensions were measured with calipers to calculate cross-sectional area and applied stress. Young’s modulus was calculated to 0.5% strain and toughness as the area under the stress-strain curve to failure. Modulus and toughness were calculated using a custom MATLAB code.

### Equilibrium water content

Small squares were cut from hydrogel slabs prepared as described in section 2.2, weighing 100–200 mg. After recording wet weights, samples were lyophilized for 72 hours, and weighed again. Equilibrium water content was calculated as (wet weight–dry weight)/ wet weight x 100%.

### Cytotoxicity

Human neonatal dermal fibroblasts (Cascade Biologics) were co-cultured with HEMA gels of select formulations: EGDMA only (12 min UV polymerization), EGDMA/VM 1/3.2 (6 min and 14 min UV polymerization), and VM only (12 and 40 min UV polymerization). Polymerization times were selected to evaluate gels at both high and low crosslinking densities. VM only and EGDMA/VM 1/3.2 formulations were selected over the other VM containing formulations, as they had the highest VM content which would be the component responsible for cytotoxicity. The human dermal fibroblasts were plated at an initial density 15,000 cells/well in a tissue culture-treated 24-well plate and cultured in Dulbecco’s Modified Eagle’s Medium (Sigma Aldrich) supplemented with 10% fetal bovine serum and 1% penicillin-streptomycin. After 14 hours, the media was aspirated and replaced with 1 mL of fresh media. Transwell inserts containing hydrogel disks cut with a 6 mm biopsy punch from a hydrogel sheet hydrated for 1 week were inserted into the wells (n = 3) and an additional 500μL of media was added to the insert. At 72 hours, wells were assayed using Vybrant MTT Cell Proliferation Assay Kit (Invitrogen). While the MTT assay measures cell metabolic activity, it is indirectly used to evaluate cell proliferation and viability [29]. Discussion of cell viability is based on the assumption that the cells evaluated have similar metabolic rates and the measured effect is due to the cell number. The 72 hour time point was selected in accordance with the ISO 10993–5:2009 standard for the in vitro assessment of cytotoxicity of medical devices.

### Statistical analysis

Error bars in figures show mean ± standard deviation. Sample n = 3 except where noted. Results were compared using one-way ANOVA with Tukey’s posthoc analysis, α = 0.05, p <0.05.

## Results and discussion

### Mechanical properties

A series of HEMA-based hydrogels were synthesized with constant mole fractions of HEMA monomer, crosslinker, and UPW. VM, AM, and AHPM were used as crosslinkers. Hydrogels were photopolymerized for different times in order to explore the range of Young’s moduli for each formulation. All mechanical testing was performed under physiological conditions (37°C in PBS). The Young’s modulus increased as a function of polymerization time (**S1 Fig**). VM and AM required much longer polymerization times than EGDMA and AHPM.

**Fig 2:**
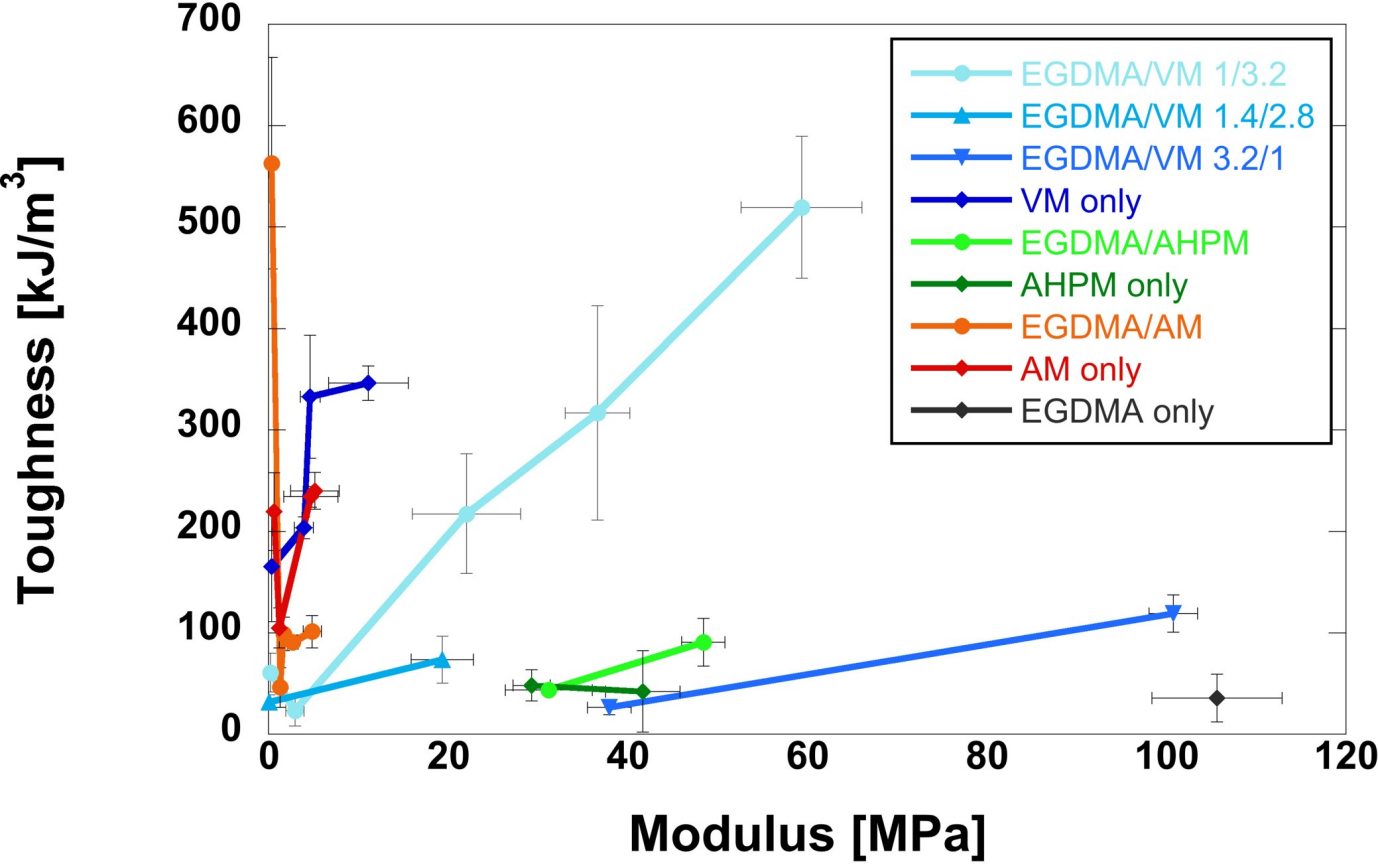
Young’s modulus vs. toughness under physiological conditions for HEMA-based hydrogels photocrosslinked at multiple time points. Chemical formulations are outlined in Table 1. UV exposure times varied across different formulations to achieve the widest possible range in modulus; exposure times for each data point are presented in **S1 Fig**.

EGDMA-crosslinked gels were stiff as synthesized [27] but were observed, qualitatively, to decrease considerably in stiffness after full hydration. However, we did not observe the same changes upon hydration in VM, AM, and AHPM gels. Rather, these gels visibly decreased in volume when hydrated and either remained the same or increased in stiffness. The highest moduli for VM and AM only gels were at or below 10 MPa. Based on these observations we chose to evaluate the effect of crosslinking HEMA with a combination of EGDMA and each new crosslinker. This was done in a 1/3.1, mol/mol, EGDMA/crosslinker ratio, as outlined in **Table 1.** As VM conferred toughness to HEMA gels while allowing for a range of moduli, we evaluated additional EGDMA/VM ratios based on the relationship between modulus and toughness at multiple UV exposure times. This relationship is displayed for all formulations in **Fig 2**. Representative stress strain curves for all formulations and exposure time points are shown in **S2 Fig**. **Fig 2**shows that AHPM crosslinked/co-crosslinked gels are stiff but not tough, and, conversely, AM crosslinked/co-crosslinked gels are tough but not stiff. Such a polarization in mechanical properties is not surprising; there is typically a tradeoff between material stiffness and toughness. AMPH gels also had a limited range of tunability. Below a UV exposure time of 10 min, resulting in a Young’s modulus of 29 ± 2 MPa, AHPM crosslinked gels were too brittle to withstand hydration. AMPH crosslinking may result in a high modulus due to the additional side group, which adds steric hindrance, reducing polymer chain mobility. One explanation for the extremely high toughness and low modulus seen for AM crosslinked gels would be an extension of that proposed by Moghadam et al. for the difference in toughness between EGDMA and TEGDMA crosslinked HEMA [3]. If the allyl double bonds were to react preferentially with each other in solution, effectively forming a new dimethacrylate crosslinker, this molecule would have a more similar length to two HEMA side chains, than either EGDMA or TEGDMA. This would also effectively reduce the number of available crosslinkers, explaining the lower Young’s modulus. It is of note that the reactivities of the various vinyl groups are not identical, meaning that preferential reactions and the resulting network structure could vary with the crosslinker incorporated.

VM incorporation is unique among the three experimental crosslinkers in its ability to enhance toughness as well as to allow for a wide range of moduli. We produced VM only gels ranging from 0.37 ± 0.03 MPa to 11.15 ± 4.45 MPa. While VM only gels tended to be tougher than EGDMA/VM gels of all ratios, they were not able to reach the full range reported for cartilage. The tensile modulus of human tracheal cartilage was reported between 1 and 20 MPa [19] and of articular cartilage between 1 and 15 MPa [20]. We produced 1/3.2, mol/mol, EGDMA/VM gels with moduli between 0.25 ± 0.03 MPa and 59.4 ± 6.7 MPa, fully spanning this range. Increasing VM content increased toughness, but decreased the Young’s modulus of the gel. Higher EGDMA content, i.e. additional methacrylate content and reduced acrylate content, was expected to increase the Young’s modulus. For applications where a modulus below 11 MPa is relevant, VM crosslinking alone will provide superior toughness. The lowest modulus EGDMA/VM gel had a toughness of 61 ± 19 kJ/m^3^, which is on par with the toughest double network and homogenous hydrogels reported in the literature [8]. EGDMA/VM gels have the additional advantage of lower UV exposure times required for polymerization. While EGDMA/VM gels were exposed for 6–14 minutes to achieve gels with the described properties, VM only gels were exposed for 12–30 minutes. Exposure to UV may be a concern when polymerizing *in vivo*.

### Equilibrium water content

**Fig 3:**
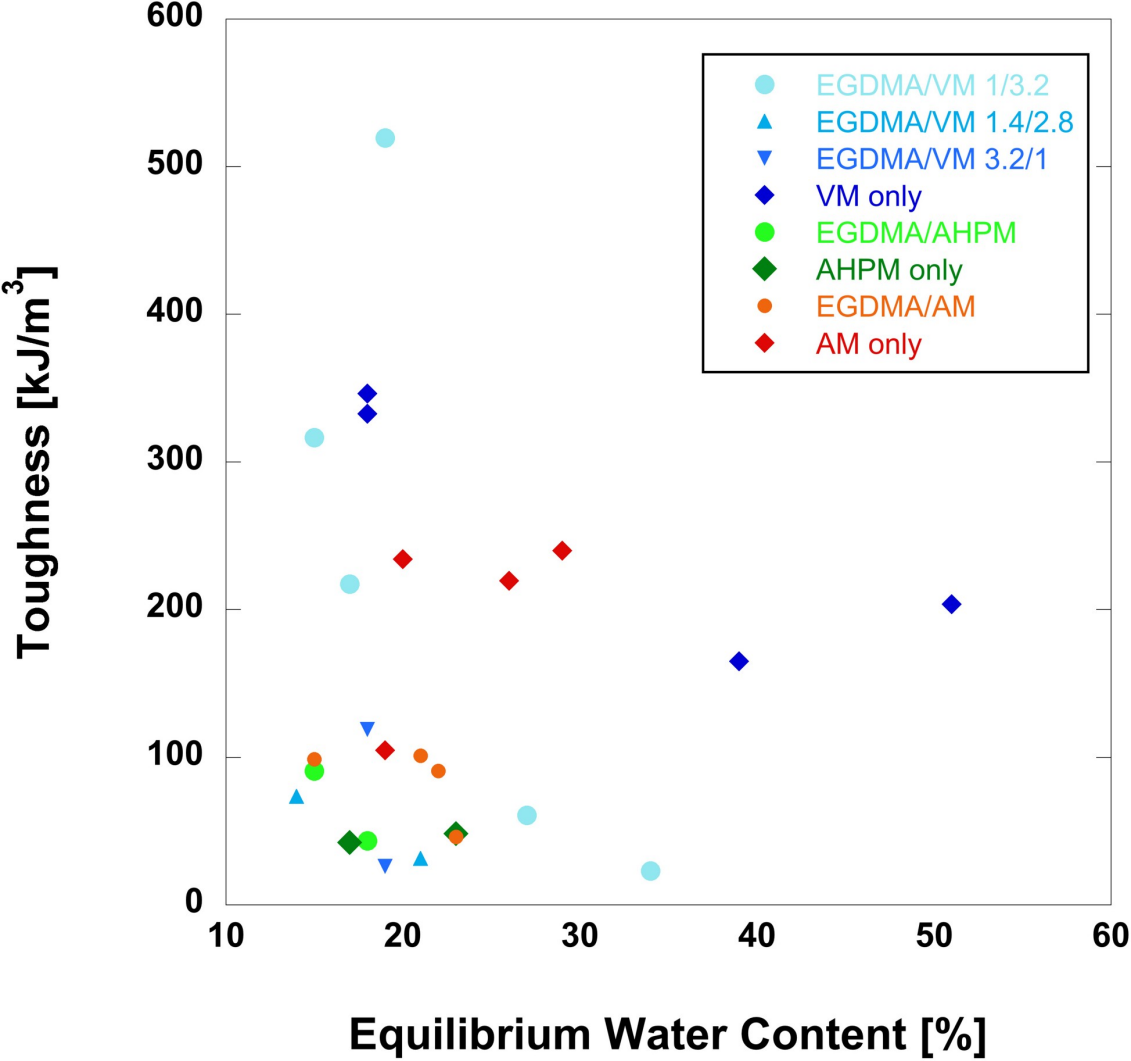
Toughness as a function of equilibrium water content for the hydrogels with formulations described in Table 1.

The equilibrium water content (EWC) was evaluated for all samples at each polymerization time (crosslinking density). The water content of swollen hydrogels is important for tissue engineering applications because swelling improves molecular transport. It would be expected for tougher samples to have lower EWC. **Fig 3**shows toughness as a function of EWC. **S3 Fig** shows toughness as a function of swelling ratio. Surprisingly, there is no clear relationship between EWC and toughness, indicating that the toughening mechanism of these heterobifunctional crosslinkers is not increased hydrophobicity. AM crosslinked and co-crosslinked gels were significantly tougher than AHPM crosslinked and co-crosslinked gels, yet have comparable or higher EWC. Notably, the VM only gels formed at lower polymerization times had significantly higher EWC than more highly crosslinked VM only gels, as would be expected. The VM only gels polymerized for 12 and 20 minutes were notably tougher than all gels crosslinked with AHPM or EGDMA/AM, despite having significantly higher EWC (≥40%). The EWC of all formulations compare favorably to published data for HEMA and tough materials. While tougher, methyl acrylate–co–methyl methacrylate networks only have an EWC of 3% (calculated as a percentage of dry weights). Liu et al. prepared macroporous HEMA hydrogels of high modulus (100–250 MPa), but even with large pores only achieved an EWC slightly higher than the values reported here of 40–60% [30].

### Cytotoxicity

**Fig 4:**
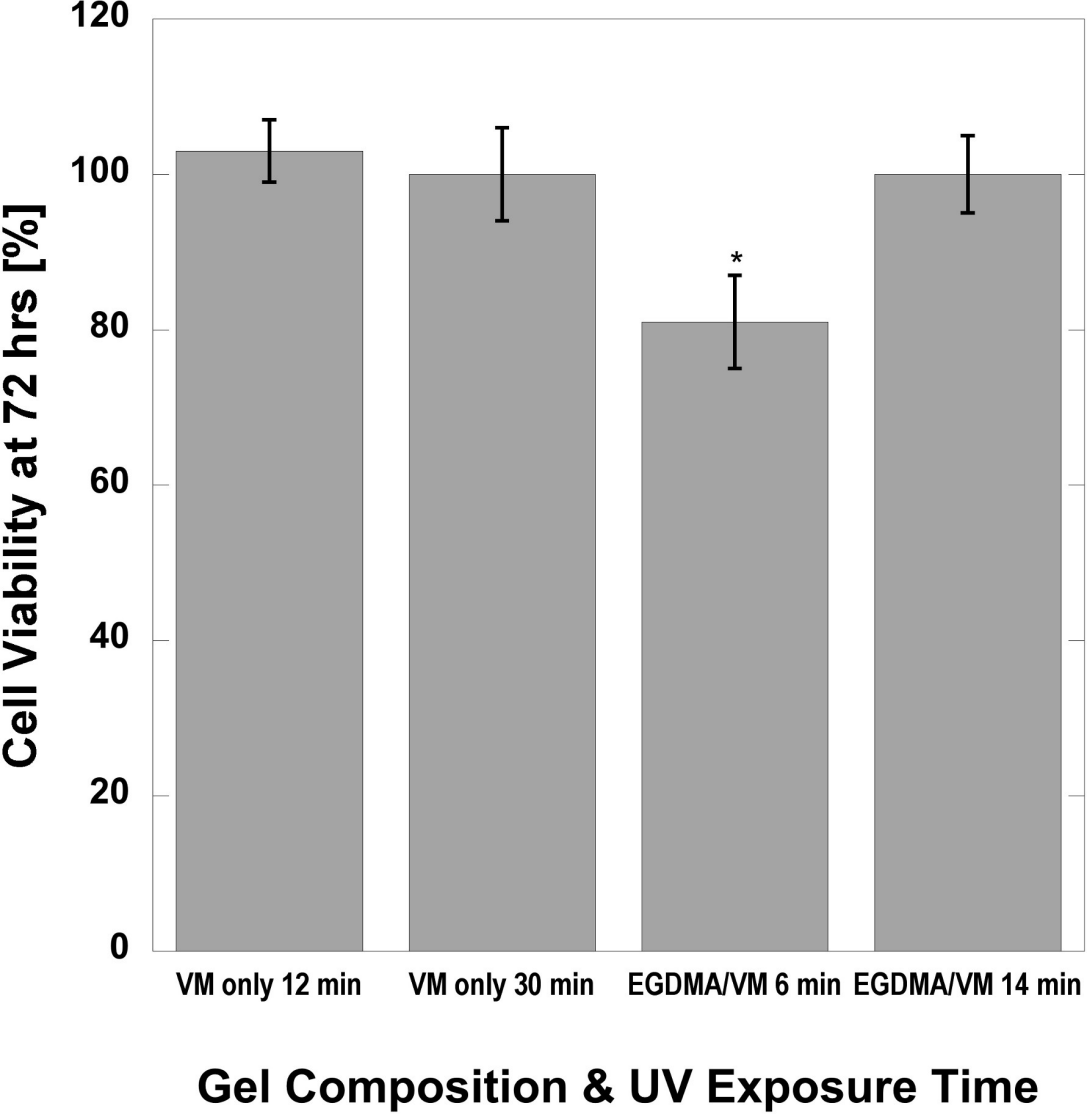
Human dermal fibroblast viability after 72 hours of co-culture with VM crosslinked and EGDMA/VM co-crosslinked HEMA hydrogels at low and high crosslinking densities.

As VM crosslinked and co-crosslinked hydrogels were deemed most suitable for load bearing tissue-engineering applications, they were evaluated for cytotoxicity. **Fig 4**shows cell viability after 3 days (72 hours) of co-culture with human dermal fibroblasts, as a percentage of EGDMA only crosslinked controls. Each formulation was evaluated for a gel with high and low UV exposure times to consider the effect of crosslinking density on the retention and leaching of toxic unreacted components. VM only was non-cytotoxic. EGDMA/VM crosslinked gels with a 6 min UV exposure time exhibited significantly lower cell viability at 72 hours. Unreacted VM content is the expected source of cytotoxicity. Considering the lack of cytotoxicity for VM only gels, we anticipate that removal of unreacted components, low modulus EGDMA/VM gels may demonstrate improved cytocompatibility. Nevertheless, cell viability exceeded 80%. In order to further investigate cytocompatibility, surface modifications to enable cell attachment will be required. Future work to create macroporous and functionalized EGDMA/VM co-crosslinked HEMA hydrogels may result in scaffolds useful for applications requiring mechanical function immediately upon implantation. While scaffold degradation is ultimately desirable for tissue engineered constructs, matching degradation and tissue maturation rates in order to maintain mechanical integrity remains a key challenge. Incorporation of enzymatically degradable crosslinks into EGDMA/VM co-crosslinked HEMA-based gels may result in a degradable hydrogel with enhanced toughness.

## Conclusions and future directions

We evaluated HEMA hydrogels crosslinked with heterobifunctional crosslinkers alone or at different ratios in an effort to achieve hydrogels with tunable Young’s moduli and toughness. Vinyl methacrylate (VM) and EGDMA/VM co-crosslinked HEMA-based hydrogels were cytocompatible, had equilibrium water content ranging from 15–54%, and exhibited mechanical properties that may be useful in load bearing applications. These hydrogels may be photopolymerized to achieve Young’s moduli under physiological conditions that encompass the full range of values reported for human cartilage.

Further analysis of EGDMA/VM co-crosslinked gels will be required to explore their full potential for biological applications. More extensive mechanical testing under physiological loading conditions under physiological loading conditions should be undertaken. Hydrogel functionalization should be pursued to enable cell attachment to the material surface and subsequent additional cytocompatibility screening. Ultimately, macro-porosity and the incorporation of degradable crosslinks could be explored, alongside functionalization, to create fully seeded, three dimensional constructs for implantation and *in vivo* biocompatibility analysis. From a materials science perspective, it would be very interesting to examine the hydrogel architecture with scanning electron microscopy. Differences in micro-architecture may illuminate the basis of the wide range of material properties achieved with crosslinker variation.

## Supporting information

S1 Fig Young’s modulus for hydrogels of all formulations outlined in Table 1 at multiple UV exposure times. (TIF)

S2 Fig Representative stress strain curves for hydrogels of all testable formulations outlined in Table 1 at multiple UV exposure times. (TIF)

S3 Fig Toughness as a function of swelling ratio, calculated as (wet weight–dry weight)/wet weight x 100%, for the hydrogels with formulations described in Table 1. (TIF)
