# Supplementary figures and images for: The effect of heterobifunctional crosslinkers on HEMA hydrogel modulus and toughness
Source: PLoS One. 2019 May 9;14(5):e0215895. doi: 10.1371/journal.pone.0215895 (PMC6508729; doi:10.1371/journal.pone.0215895)

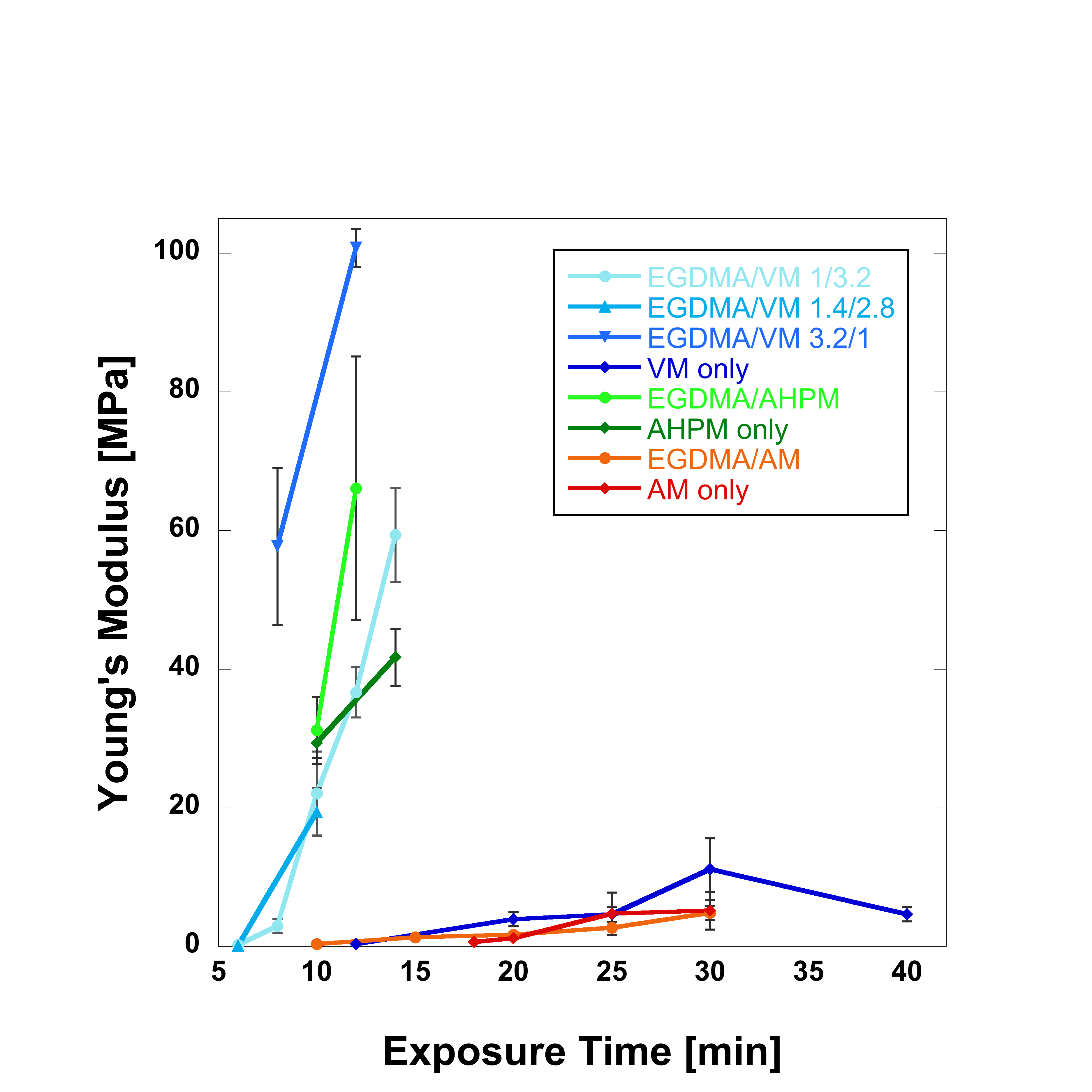

Supplement: S1 Fig — (TIF) [file pone.0215895.s001.tif]

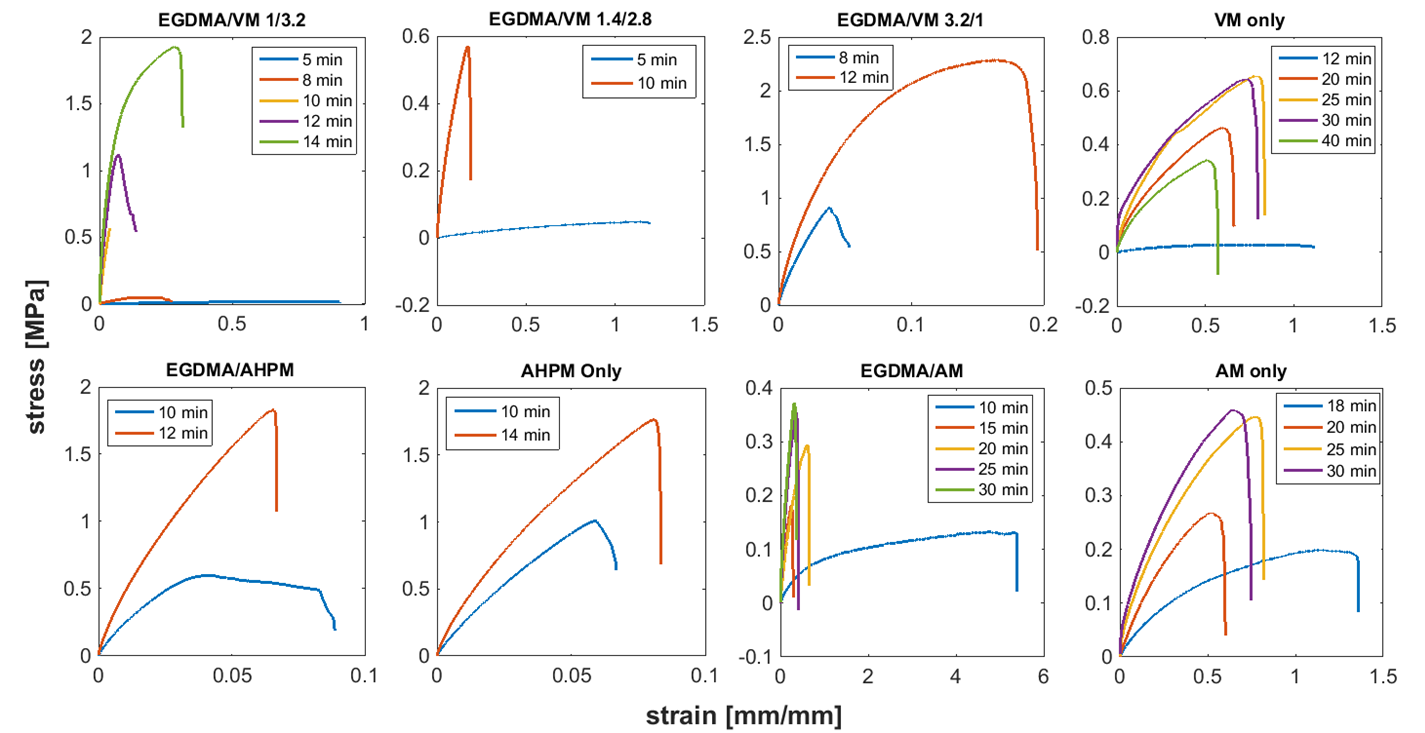

Supplement: S2 Fig — (TIF) [file pone.0215895.s002.tif]

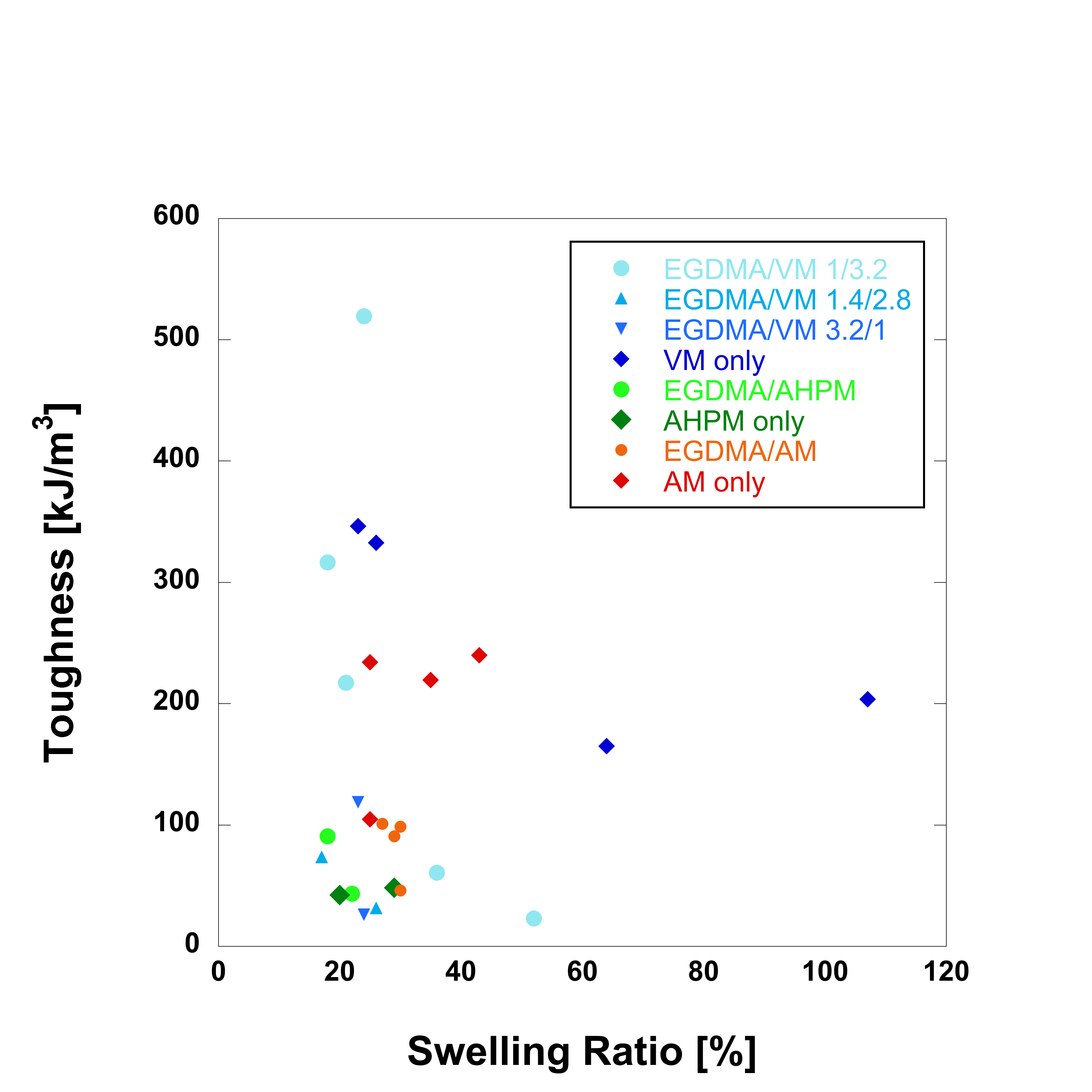

Supplement: S3 Fig — (TIF) [file pone.0215895.s003.tif]
